# Nosocomial outbreak of coronavirus disease in two general wards during the initial wave of the pandemic in 2020,  Tokyo, Japan

**DOI:** 10.5365/wpsar.2022.13.1.906

**Published:** 2022-03-24

**Authors:** Naoya Sakamoto, Masayuki Ota, Tomoko Takeda, Atsushi Kosaka, Takuya Washino, Sentaro Iwabuchi, Minako Beppu, Itaru Nishiduka, Tamano Matsui, Motoi Suzuki, Fukumi Nakamura-Uchiyama

**Affiliations:** a Department of Infectious Diseases, Tokyo Metropolitan Bokutoh General Hospital, Tokyo, Japan.; b Field Epidemiology Training Program, National Institute of Infectious Diseases, Tokyo, Japan.; c Sumida Public Health Center, Tokyo, Japan.; d Center for Surveillance, Immunization, and Epidemiologic Research, National Institute of Infectious Diseases, Tokyo, Japan.

## Abstract

**Objective:**

Coronavirus disease 2019 (COVID-19), caused by severe acute respiratory syndrome coronavirus 2 (SARS-CoV-2), was first reported in China and subsequently spread worldwide. In Japan, many clusters occurred during the first wave in 2020. We describe the investigation of an early outbreak in a Tokyo hospital.

**Methods:**

A COVID-19 outbreak occurred in two wards of the hospital from April to early May 2020. Confirmed cases were individuals with laboratory-confirmed SARS-CoV-2 infection linked to Wards A and B, and contacts were patients or workers in Wards A or B 2 weeks before the index cases developed symptoms. All contacts were tested, and cases were interviewed to determine the likely route of infection and inform the development of countermeasures to curb transmission.

**Results:**

There were 518 contacts, comprising 472 health-care workers (HCWs) and 46 patients, of whom 517 were tested. SARS-CoV-2 infection was confirmed in 42 individuals (30 HCWs and 12 patients). The proportions of SARS-CoV-2 infections in HCWs were highest among surgeons, nurses, nursing assistants and medical assistants. Several HCWs in these groups reported being in close proximity to one another while not wearing medical masks. Among HCWs, infection was thought to be associated with the use of a small break room and conference room.

**Discussion:**

Nosocomial SARS-CoV-2 infections occurred in two wards of a Tokyo hospital, affecting HCWs and patients. Not wearing masks was considered a key risk factor for infection during this outbreak; masks are now a mandated countermeasure to prevent the spread of SARS-CoV-2 infection in hospital settings.

Coronavirus disease 2019 (COVID-19), caused by severe acute respiratory syndrome coronavirus 2 (SARS-CoV-2), was first reported in Wuhan City, China, in December 2019 and rapidly spread worldwide. (*1*) Nosocomial SARS-CoV-2 outbreaks have been reported in several countries, including Australia, (*2*) China, (*3*, *4*) Germany (*5*) and Japan. (*6*-*8*)

On 7 April 2020, a patient who had previously been admitted to a Tokyo hospital due to an exacerbation of chronic heart failure developed a fever and dyspnoea 10 days after discharge and was subsequently diagnosed with COVID-19. At the same time, a member of the cleaning staff from the same ward as the patient (Ward A) was referred to the emergency department with fever and dyspnoea and was subsequently diagnosed with COVID-19. On 14 April 2020, two patients, a nurse and a nursing assistant in Ward B became febrile. The two health-care workers (HCWs) were also diagnosed with COVID-19.

This report summarizes the outbreak investigation conducted into the COVID-19 cases in Wards A and B by the hospital infection control team and public health centre staff during the early stages of the COVID-19 pandemic, a period of low community transmission in Tokyo.

## Methods

### Setting

The hospital in Tokyo is a tertiary care facility with 765 beds and 39 subspecialties, including an infectious diseases department. Ward A is a general ward for patients with heart or renal disease, and Ward B is a general ward for surgery, gynaecology and gastrointestinal disease patients. During the 3 months preceding April 2020, the occupancy rate in the hospital’s 719 general beds, including 32 intensive care unit beds, ranged from 80% to 85%.

### Outbreak investigation and laboratory methods

Confirmed cases were defined as individuals with a positive SARS-CoV-2 result from real-time reverse transcription polymerase chain reaction (RT–PCR) testing of a nasopharyngeal sample or sputum sample, regardless of whether they were symptomatic, as per the World Health Organization’s interim guidance for COVID-19 surveillance. (*9*) A cluster was defined as more than two epidemiologically linked cases, such as people who were on the same ward at the same time.

The two clusters of COVID-19 in Wards A and B were reported to the public health centre on 20 April 2020, and the hospital and public health centre requested assistance in investigating the outbreak from the Ministry of Health, Labour and Welfare in Japan. The assistance provided by the Ministry included developing infection prevention and control measures and interviewing hospital staff to assess their use of personal protective equipment, the break room and conference room; the nursing system – for example, how many patients each nurse was responsible for and how teams of nurses worked; and frequency of patient contact among HCWs. Interviews were conducted by an experienced interviewer, but the interviewer did not use a standardized questionnaire.

As the source of the virus within the hospital was unclear and there was a possibility of additional undetected cases, we used a broad definition of a contact. A contact was defined as an individual who was hospitalized or worked in Ward A or B 2 weeks before the index cases developed symptoms. This group included discharged patients from both wards and patients who had been transferred from Ward A or B to other wards.

All contacts were tested using RT–PCR. A nasopharyngeal swab or sputum sample was collected from all contacts initially, and if the first test result was negative, contacts were retested if they developed new or persistent symptoms. If a HCW tested positive, all HCWs using the same break room or conference room were considered contacts and tested. If a contact tested positive, all patients in the same room as a patient and all HCWs who had had contact with the patient were also considered contacts and tested.

Patients in Wards A and B were observed for symptoms for 14 days after outbreak control measures were implemented, and HCWs were followed up for 14 days from their final exposure to an index case or cluster.

## Results

### Outbreak description

From 7 April to 3 May 2020, the time from the first case until the last contact was tested, 518 contacts were identified (472 HCWs and 46 patients). The HCWs included 107 doctors, 62 nurses and 303 other medical personnel. All but one contact (471 HCWs and 46 patients) underwent RT–PCR testing. **Table 1** summarizes the outbreak investigation of the HCWs.

**Table 1:** Number and proportion of health-care workers by test result in the investigation of a COVID-19 outbreak in two wards of a Tokyo hospital, Japan, 2020

Working ward and occupation	Health-care workers					
	Total no.	No. defined as contacts	No. tested	No. positive tests		Positive test (%)
				Symptomatic	Asymptomatic	
Ward A						
Nurse	34	34	34	0	4	11.8
Doctor	-	-	-	-	-	-
Cardiologist	14	14	14	0	0	0
Nephrologist	9	9	9	0	0	0
Cardiac surgeon	3	3	3	0	0	0
Other doctor	9	9	9	0	0	0
Ward B						
Nurse	28	28	28	4	0	14.3
Doctor	-	-	-	-	-	-
Surgeon	23	23	23	1	6	30.4
Gynaecologist	22	22	22	0	1	4.5
Gastroenterologist	20	20	20	0	1	5.0
Other doctor	7	7	7	0	0	0
Unspecified ward^a^						
Nursing assistant	29	29	29	3	2	17.2
Clerk	32	32	31	1	2	9.7
Medical worker	81	76	76	2	0	2.6
Radiology technologist	51	49	49	0	1	2.0
**Other occupations^b^**	155	117	117	1	1	1.7
**Total**	**517**	**472**	**471**	**12**	**18**	**6.4**

^a^ This category includes those who worked on all hospital wards – that is, they worked on either or both wards A and B – and those who tested positive during contact tracing.

^b^ Workers in this category included pharmacists, rehabilitation therapists, laboratory technicians and cleaning staff.

**Fig. 1:**
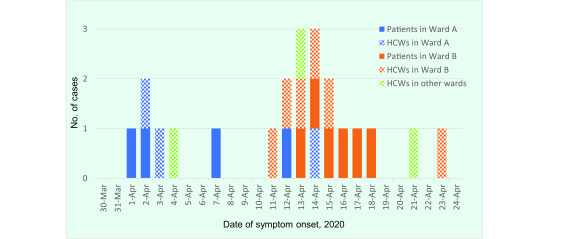
Epidemic curve of confirmed symptomatic cases of COVID-19, by date of symptom onset during an outbreak in two wards in a Tokyo hospital, Japan, 2020 (n = 22)

A total of 42 people had positive RT–PCR tests: 30 HCWs and 12 patients. Of the 30 positive HCWs, 12 were symptomatic (nine from Ward A or B and three who reported having contact in the break room with workers from Ward A or B who tested positive) and 18 were asymptomatic. Of the 12 positive patients, 10 were symptomatic and two were asymptomatic. The SARS-CoV-2 infection rate among surgeons in Ward B was 30.4% (7/23), for nursing assistants the rate was 17.2% (5/29), for nurses in Ward A it was 11.8% (4/34), for nurses in Ward B it was 14.3% (4/28) and among clerks it was 9.7% (3/31), compared with rates of 0–5% among the other occupational groups, which included, for example, pharmacists, laboratory technicians and cleaning staff (**Table 1**). The epidemic curve of symptomatic cases suggests that the outbreak started in Ward A and spread to Ward B within a week (**Fig. 1**).

[**insert** Figure 1]

At interview, some surgeons reported not wearing masks during their biweekly conferences in a small conference room and other HCWs reported using the small break room without masks.

### Outbreak management

After the two clusters of COVID-19 were recognized in the two wards, new admissions were stopped on Ward A on 14 April 2020, in Ward B on 16 April 2020 and hospital-wide on 21 April 2020. All patients who met discharge criteria, except those in Wards A and B, were discharged. On 18 April 2020, all medical services were suspended, including at the tertiary care centre. However, 24-hour emergency services for patients not requiring admission, the perinatal medical centre and psychiatric emergency centre remained open. Visits by family members had been restricted from early March 2020 (i.e. they were limited to family members who had been asymptomatic within the previous 2 weeks, a maximum of 15 minutes per visit, and only one visitor per patient), and visits were completely banned after 27 April 2020. All COVID-19 cases in the hospital were transferred to a dedicated ward, and patients from Wards A and B with negative RT–PCR results were isolated in Ward A. Environmental cleaning was conducted in all wards.

Break room use by HCWs was modified so that fewer people used the rooms; HCWs were advised not to sit facing each other; and partitions were provided for when they had to face each other. Before starting work each day, staff were asked about symptoms and had their temperature checked. The infection control team provided education to HCWs about control measures. These measures were implemented for approximately 1 month, and there were no new laboratory-confirmed cases after 28 April 2020. The hospital resumed regular services on 18 May 2020, with a dedicated ward for patients suspected to have COVID-19 (i.e. patients with acute respiratory failure or fever of unknown origin).

## Discussion

This report describes a COVID-19 outbreak in a tertiary care hospital in Tokyo during the first phase of the pandemic. Clusters of cases were reported from two general wards, with the infection spreading among HCWs. The source of SARS-CoV-2 infection in each ward was not identified.

The infection rate of SARS-CoV-2 for HCWs was highest among surgeons, nurses and clerks. Several surgeons reported holding twice-weekly conferences in the small conference room, and nurses reported using a small break room, both of which may have contributed to transmission, as has been reported previously. (*2*) There were 18 asymptomatic cases in HCWs who also may have contributed to the spread of COVID-19 in the hospital while they were unknowingly infectious, (*10*-*12*) especially among those who gathered in close proximity without wearing a medical mask. Appropriate mask use by HCWs can prevent the spread of SARS-CoV-2 in hospitals and allow for better infection control. (*13*-*15*) Although self-quarantine, universal mask use and physical distancing are now standard practice, (*4*, *16*) these were not universal during the early phase of the pandemic. It is also possible that some infections in HCWs might not have been transmitted in the hospital but may have been community-acquired, although the rate of community-acquired infection was relatively low at the time.

This study has some limitations. A standardized questionnaire was not used during interviews with contacts; therefore, the data collected did not allow for robust analysis of the use of break rooms and conference rooms; personal protective equipment, such as gloves and gowns; handwashing; the performance of medical procedures for inpatient care; and the extent and timing of exposure to infected patients. Also, the approximately 2-week delay in collecting information is likely to have resulted in some recall bias. Contact tracing was limited to the hospital setting, and there was no contact tracing among outpatients or visitors. However, we consider it unlikely that SARS-CoV-2 infections were introduced by outpatients or visitors because outpatients usually do not enter the wards and there were restrictions on visitors. Finally, we were unable to standardize the timing of testing after exposure and the retest protocol for contacts who were negative on initial testing, and this could have resulted in false-negative cases.

In summary, this outbreak investigation documents nosocomial SARS-CoV-2 infection among HCWs and patients that occurred in two hospital wards during the initial wave of the COVID-19 pandemic in 2020 in an area with a low rate of community-acquired infection and before vaccines were available. Extensive contact tracing was conducted with high testing coverage only of contacts within the hospital setting. Because nosocomial infections can spread from asymptomatic or  presymptomatic individuals to unmasked HCWs, stringent infection prevention and control measures are required to prevent hospital-based outbreaks; these measures include wearing masks and avoiding close contact when not wearing medical masks in small rooms.
